# *Cryptococcus neoformans* meningitis in kidney transplant recipients: A diagnostic and therapeutic challenge

**DOI:** 10.1016/j.mmcr.2021.04.005

**Published:** 2021-05-04

**Authors:** Julien Gras, Yanis Tamzali, Blandine Denis, Maud Gits-Muselli, Stéphane Bretagne, Marie-Noëlle Peraldi, Jean-Michel Molina

**Affiliations:** a Infectious Diseases Department, APHP, Saint-Louis Hospital, Paris, France; b INSERM U944, “Cellular Biology of Viral Interactions” Team, Université de Paris, France; c Parasitology and Mycology Department, APHP, Saint-louis Hospital, Paris, France; d Nephrology and Kidney Transplant Department, APHP, Saint-louis Hospital, Paris, France

**Keywords:** Cryptococcosis, Meningitis, Kidney transplantation, Graft function, Early diagnosis

## Abstract

Cryptococcosis is the third most common invasive fungal infection in solid organ transplant recipients. We describe three cases of neuro-meningeal cryptococcosis occurring among kidney transplant (KT) patients, and discuss the diagnostic and therapeutic challenges in this context.

Median time from KT to infection was 6 months [range: 3–9]. The most common clinical manifestations at diagnosis were fever (2/3), headache (2/3), and confusion (2/3); none had extra-neurological involvement. CrAg was positive in all cases at diagnosis both in serum and cerebrospinal fluid (CSF). For two patients, analysis of previous samples showed that CrAg was detected in plasma up to 4 weeks before diagnosis. All patients received induction treatment with liposomal amphotericin-B (L-AmB) and flucytosine for a median duration of 10 days [range: 7–14], followed by fluconazole maintenance therapy. Acute kidney injury secondary to L-AmB therapy was observed in only one case, but all patients had a tacrolimus overdose following initiation of maintenance therapy due to drug-drug interactions between fluconazole and tacrolimus.

Among KTR, early detection of *Cryptococcus* meningitis using serum CrAg is possible. Close monitoring of renal function during treatment is essential due to the nephrotoxicity of L-AmB, but also drug-drug interactions between fluconazole and calcineurin inhibitors.

## 1 Introduction

Cryptococcosis is a severe fungal infection which primarily affects immunocompromised patients with cellular immune deficiency, in particular HIV-positive individuals in whom it is associated with a high mortality rate [1]. With the implementation of highly-active antiretroviral therapy (HAART), cryptococcosis is nowadays more often reported in HIV-negative patients, especially solid organ transplant (SOT) recipients which account for 20–60% of cryptococcosis cases among HIV-negative individuals [2]. Cryptococcosis represents the third most common fungal infection in SOT recipients after invasive candidiasis and aspergillosis [3], with approximately half of the cases involving central nervous system [4].

Despite the improvement of diagnostic tools and treatment protocols, cryptococcosis in SOT recipients remains a diagnostic and therapeutic challenge, and still causes significant morbidity and mortality [5]. Between 2006 and 2018, the mortality of cryptococcosis three months after diagnosis was 20–25% among SOTR compared to 14.3% among HIV-positive individuals according to data from the French national reference center for invasive mycoses. Several factors can account for the high burden of this infection among transplant recipients. First, clinical presentation of cryptococcosis in SOT recipients differs from HIV-positive individuals with less frequent involvement of CNS disease [6]. Second, the diagnosis is frequently delayed due to the variety of clinical symptoms and the absence of a systematic screening strategy. Finally, antifungal treatment is complicated by the nephrotoxicity of Liposomal Amphotericin-B (L-AmB) in patients with frequent impaired renal function [7], but also by potential drug-drug interactions between fluconazole and calcineurin inhibitors (CNI) [8].

We herein report three cases of cryptococcal meningitis in kidney transplant recipients in a French tertiary hospital and discuss the clinical presentation, biological diagnosis, therapeutic management, and renal outcome after a review of the literature.

## 2 Case presentation

Between 1995 and 2019, in the APHP (Assistance Publique des Hôpitaux de Paris)-Saint Louis Hospital (Paris, France), three cases of neuro-meningeal cryptococcal infection were recorded among KT recipients. During the same period, 2549 kidney transplants were performed, leading to a frequency of 0.12% for cryptococcal meningitis in our center. The main characteristics of the three cases of cryptococcal meningitis are described in Table 1.

**Table 1:** Characteristics of the three cases of cryptococcal meningitis in KT recipients.

Variable	1	2	3
**Age at transplantation** (years)	45	67	47
**Country of origin**	France	France	Sri Lanka
**Underlying comorbid disease**			
Diabetes mellitus	No	Yes	No
HIV-infection	No	Yes	No
**Characteristics of kidney transplantation**			
Underlying nephropathy	IgA nephropathy	IgA nephropathy	Unknown
Immunosuppressive treatment before transplantation	Corticosteroids	No	No
Duration of dialysis before transplantation (months)	0	6	36
Induction therapy			
Anti T-cell antibodies	Yes	Yes	Yes
High-dose IV corticosteroids	Yes	Yes	Yes
**Time between KT and cryptococcal infection** (months)	9	6	3
**Clinical symptoms at diagnosis**			
Fever	No	Yes	Yes
Neurological signs	Yes	Yes	Yes
Extra-neurological involvement	No	No	No
**CSF analysis**			
Cell count (/μL)	175	2	14
Protein level (g/L)	1.37	0.46	0.32
Glucose level (g/L)	0.14	1.2	0.5
Opening pressure (cm H_2_O)	10	40	52
India ink staining on direct examination	Positive	Positive	Positive
Cryptococcal Ag testing	Positive (1/16)	Positive (1/4090)	Positive (1/64)
Mycological culture	Negative	Positive	Positive
**Blood samples analysis**			
Serum cryptococcal antigen testing	Positive (1/64)	Positive (1/65536)	Positive (1/512)
Mycological blood cultures	Negative	Positive	Positive
**Antifungal therapy**			
Induction therapy (L-AmB + Flucytosine)			
Duration (days)	14	7	10
Acute kidney injury	No	No	Yes
Fluconazole maintenance therapy			
Initial dose (mg per day)	400	600	600
Drug-drug interactions with CNI	Yes	Yes	No
**Modifications in the immunosuppressive regimen**			
Corticosteroids	Unchanged	Unchanged	Interrupted
Mycophenolate mofetil	Switch to AZA	Interrupted	Interrupted
Tacrolimus	50% dose reduction	50% dose reduction	80% dose reduction

Abbreviations: Ag, antigen; AZA: Azathioprine; CSF, cerebrospinal fluid; IV, intravenous; KT: Kidney transplantation; L-AmB: Liposomal amphotericin B; NA: not available.

### 2.1 Case 1

The first case was a French 45-year-old man with no history of travelling abroad, who was diagnosed with IgA-nephropathy in 2009. Kidney dysfunction progressed to end-stage disease despite corticosteroid treatment initiated in 2013, and the patient was finally grafted in January 2015 from a living donor. Induction therapy included anti-T cell antibodies and high-dose intra-venous (IV) corticosteroids; maintenance treatment consisted in conventional triple immunosuppressive therapy (CNI, MMF, and oral corticosteroids with slow tapering). Initial outcome after transplantation was favorable with immediate kidney function recovery and no infectious complication. Six months later, in June 2015, the patient experienced recurrent headaches which progressively worsened. On admission in September 2015, the patient had no fever and neurological examination was normal. The lumbar puncture showed neutrophilic hypoglycorachic meningitis and a normal CSF opening pressure; India ink examination of CSF was positive but cultures remained sterile. Cryptococcal antigen was positive both in the CSF (1/16) and the serum (1/64), Induction therapy with L-AmB (3 mg/kg QID) and flucytosine (100 mg/kg) was initiated with rapid clinical and mycological efficacy (negativisation of CrAg both in serum and CSF). Initial tolerance was good as serum creatinine level remained stable. At the same time, immunosuppressive regimen was lowered with the interruption of MMF (replaced by azathioprine). After 2 weeks, antifungal treatment was switched to fluconazole maintenance therapy (800 mg on day one followed by 400 mg per day). Three days after the switch to fluconazole, we observed a rise in tacrolimus level (from 8.2 to 24 mg/L), followed by acute kidney injury (AKI) as serum creatinin levels rose from 95 μmol/L to 175 μmol/L. Kidney function slowly came back to normal after the adaptation of tacrolimus levels, and no alteration was further observed under fluconazole maintenance therapy. The outcome after three years under fluconazole secondary prophylaxis (100 mg per day) was favorable with a stable graft function, and no rejection episode despite immunosuppression modulation.

### 2.2 Case 2

The second case was a 67-year-old man, HIV-positive since 1995, with a history of diabetes mellitus and a Hodgkin lymphoma diagnosed in 2007 in complete remission after chemotherapy. He developed end-stage renal failure secondary to an IgA-nephropathy and Cacchi-Ricchi related disease. He had no history of living or travelling abroad. In October 2017, after six months of peritoneal dialysis, he underwent a KT from a deceased donor with a high immunological risk induction protocol including anti-T cell antibodies and high-dose IV corticosteroids. In the first months after KT, creatinine levels increased due to vascular toxicity of CNI, but there was no immediate infectious complication with an excellent virologic control of the HIV infection despite persistent lymphopenia (undetectable HIV viral load, CD4 34/μL).

Six months after KT, in April 2018, the patient developed progressive asthenia and weight loss for which he was admitted in hospital. On admission, physical examination showed fever, and abnormal mental status with confusion. A lumbar puncture was performed and revealed an elevated opening pressure (40 cm H_2_0), but no pleocytosis. CSF analysis was positive for India ink examination and for cryptococcal antigen testing (1/4090); CSF samples grew positive for *Cryptococcus neoformans* var. *grubii* (serotype A) after three days. Cryptococcal antigen tested also positive in the serum, and two blood cultures were positive for *C. neoformans*. Despite early initiation of L-AmB and flucytosine (and immediate interruption of MMF), the patient's neurological status deteriorated and presented seizures for which he was transferred to intensive care unit. Mental status and CSF opening pressure were normalized after three consecutive lumbar punctures. Antifungal treatment was switched to oral fluconazole (600 mg per day) after only one week of induction therapy given the clinical improvement and cytopenia due to 5-FC, and despite the absence of AKI under L-AmB. Following the initiation of fluconazole maintenance therapy, there was a rise in tacrolimus level (from 7.2 to 12.7 mg/L after 5 days) without any concomitant overdose of fluconazole. However, kidney function remained stable thanks to a quick reduction of both tacrolimus and fluconazole dose (from 600 to 200 mg/day after seven days). Clinical outcome was favorable under fluconazole maintenance therapy during 10 weeks, followed by secondary prophylaxis (100 mg per day).

### 2.3 Case 3

The third case was a 47-year-old man born in Sri Lanka, who arrived in France in 2004 where he was diagnosed with end-stage chronic renal failure of undetermined cause. Hemodialysis was initiated in 2014, and he underwent a kidney transplantation in December 2017 with a high immunological risk induction protocol, with complete kidney function recovery after renal replacement therapy. Three months later, at the beginning of March 2018, the patient presented with headaches, followed by the appearance of photophobia and phonophobia, and fever. On hospital admission, the patient was febrile (38.3 °C) and confused. The lumbar puncture revealed lymphocytic hypoglycorachic meningitis with encapsulated yeasts on direct microscopy using India ink examination, and culture came back positive for *Cryptococcus neoformans* var. *grubii* (serotype A). Cryptococcal antigen tested positive both in the serum (1/512) and the CSF (1/64). Neurological imaging (MRI) showed diffuse leptomeningitis with cerebellar vasculitis. Antifungal induction therapy with L-AmB (3 mg/kg QID) and flucytosine (100 mg/kg QID) was initiated. Due to the persistence of clinical signs of elevated intracranial pressure, lumbar punctures were repeated for CSF drainage and IV corticosteroid added on day 8. After ten days of antifungal treatment, L-AmB was discontinued due to the rise of serum creatinine level and drug-induced tubulopathy, and induction therapy was switched to fluconazole (600 mg per day). Despite tacrolimus dose reduction at the time of fluconazole initiation to avoid drug-drug interactions, we observed a rise in tacrolimus levels from 7.5 to 12 mg/L. Both fluconazole and CNI dose were reduced (with interruption of MMF), avoiding further increase in serum creatinine levels. Clinical outcome was favorable with neurological improvement to baseline at the time of discharge. Kidney function improved a few days after the discontinuation of L-AmB, and came back to normal two months later, after an episode of acute pyelonephritis associated with transient AKI.

### 2.4 Cryptococcal antigen testing

For two of the three cases (Patients #2 and #3), stored plasma samples were available between kidney transplantation and diagnosis to perform cryptococcal antigen testing using Biosynex CryptoPS Immunochromatographic test® (ICT test) (Table 2). For both patients, CrAg testing on plasma was positive when they started developing neurological symptoms, in a median time of one month prior to definitive diagnosis.

**Table 2:** Results of cryptococcal antigen testing (ICT test) for two of the three cases of cryptococcal meningitis on available plasma samples at the time of diagnosis and on previous visits.

Patient number	Time	Plasma Cryptococcal Ag ICT	
		(Biosynex CryptoPS Immunochromatographic test®)	
		Result	Index
2	W-12	Negative	
	W-4	Positive	T1
	W-2	Positive	T1 + T2
	Diagnosis	Positive	T1 + T2
3	W-8	Negative	
	W-4	Positive	T1
	W-1	Positive	T1
	W-1	Positive	T1 + T2
	Diagnosis	Positive	T1 + T2

Abbreviations: Ag, antigen; ICT, immunochromatography testing; W, week; Biosynex CryptoPS Immunochromatographic test® on plasma is a semi-quantitative test which is considered negative (no band), or positive with one (T1) or two bands (T1 + T2) according to the concentration of *Cryptococcus* capsular antigen (T1 ≥ 25 ng/ml; T2 ≥ 2.5 μg/L).

## 3 Discussion

In this case series, we report the medical record of three kidney transplant recipients diagnosed with cryptococcal meningitis, who successfully treated with less than 14 days’ induction therapy including L-AmB. We show that CrAg was positive one month prior to diagnosis in 2 of the three cases, and advise clinicians to be careful regarding potential drug-drug interactions between fluconazole and tacrolimus following initiation of maintenance therapy (which can lead to impaired kidney function).

In our study, the frequency of cryptococcal disease was 0.12%, similar to those observed in previous studies focusing on kidney transplant recipients [9]. Interestingly, the three cases were all diagnosed very late regarding the duration of the study period (19995–2019). This is probably due to the recent introduction of more potent immunosuppressive treatments and improved ascertainment of the cryptococcal infection in this population. The incidence of cryptococcosis is low among SOTR, ranging from 0.2 to 4.1% according to the country of origin, the type of organ transplanted and the duration of follow-up [2,4,5], and KTR seem to be at lower risk compared to liver or lung transplant recipients, probably due to the lower intensity of immunosuppressive therapy. Several risk factors for cryptococcosis have already been identified in SOTR: an older age, social precarity, underlying comorbidities (diabetes mellitus, HIV-infection), but also the type of immunosuppressive regimen (induction therapy with alemtuzumab [anti-CD52] or anti-thymocyte globulin, maintenance treatment with corticosteroids) [5]. The three patients described in our study exhibited one or several of these risk factors, emphasizing the need to be watchful for *Cryptococcus* sp. infection in the most at-risk KTR especially if they develop symptoms compatible with the diagnosis.

Cryptococcosis in SOT recipients can be revealed by a wide variety of symptoms including skin lesions (especially non healing ulcers), asymptomatic pulmonary nodule or pneumonias, osteoarticular involvement, and neurological signs [1]. Meningitis is frequent in immunosuppressed individuals (ranging from 44% to 97% according to the series), but is not always associated with clinical symptoms [6]. Among the three cases of cryptococcal meningitis we describe, all the patients had neurological signs, ranging from isolated headaches (patient #1), to confusion and seizures related to intracranial hypertension (patient #2). Other neurological symptoms reported in the literature are nucheal stiffness (14–20%), and visual impairment (up to 10% of the cases) [10]. Interestingly, although blood cultures were positive in two out of three cases, none of them had evidence of extra-neurological involvement following extensive investigations (including thorough cutaneous examination and pulmonary CT-scan).

None of the clinical signs of cryptococcal meningitis are specific, and definitive diagnosis is made by the lumbar puncture which usually shows elevated opening pressure, lymphocytic pleiocytosis, hyperproteinorachia, and hypoglycorachia. India ink examination is positive in most cases (38–93%), and confirmation is obtained by culture. Cryptococcal antigen is the most sensitive test for the diagnosis of CNS infection both in the CSF (up to 100% sensitivity) and in the serum (above 85% sensitivity) [4]. All three cases described here tested positive for cryptococcal antigen both in blood and CSF with high titers. Interestingly, we showed using stored plasma samples that cryptococcal antigen was positive in plasma up to four weeks before the date of diagnosis, when patients were mildly symptomatic. Among HIV-positive patients with advanced disease, serum cryptococcal antigen testing followed by preemptive fluconazole therapy has been associated with a decreased incidence of cryptococcal meningitis and improved survival [11]. Although routine screening for cryptococcal infection may not be beneficial in SOTR due to the low prevalence of the disease in this population, our results suggest that prompt testing using serum cryptococcal antigen even with minor neurological symptoms could allow a more early diagnosis of cryptococcal meningitis.

Following definitive diagnosis, current guidelines recommend that SOT recipients with cryptococcal CNS disease should receive induction regimen with L-AmB (3–4 mg/kg per day IV) plus flucytosine (100 mg/kg per day divided in 4 doses) for at least 2 weeks, followed by maintenance therapy with fluconazole (400–800 mg per day orally) for 8 weeks, and secondary prophylaxis (low-dose fluconazole for 6–12 months) [12]. In KTR, treatment is complicated by the nephrotoxicity of L-AmB and potential drug-drug interactions involving CNI and fluconazole, which can lead to renal function impairment [7,8]. In our study, we observed an increase in serum creatinine levels after the initiation of L-AmB therapy (associated with drug-induced tubulopathy) in only one patient. Of note, two of the three cases received less than two weeks of L-AmB (7 days for patient #2 due to 5-FC induced cytopenia, and 10 days for patient #3 due to L-AmB nephrotoxicity), but both had a good outcome. In resource-constrained settings, shorter duration of induction therapy with L-AmB has been showed to have a more favorable side-effect profile than a two week regimen for the treatment of HIV-associated cryptococcal meningitis, with no reduction in the rate of fungal clearance [13]. Although shorter treatment regimens have not been evaluated in kidney transplant recipients, they could be beneficial in well-chosen circumstances to avoid renal failure and potential graft loss.

Besides the direct nephrotoxicity of L-AmB, another potential cause of renal impairment during cryptococcosis antifungal therapy is the occurrence of drug-drug interactions between azoles and immunosuppressive agents, that we observed in all the patients of our study. Azoles are potent inhibitor of the cytochrome P450 3A system which metabolizes tacrolimus. Fluconazole can lead to increased tacrolimus concentrations by up to four-fold in SOT recipients, which may then cause acute renal impairment in a dose-dependent manner [14]. This is indeed what we recorded in all three patients, in whom tacrolimus levels significantly increased after the switch to fluconazole. However, renal failure only occurred in patient #1, whereas for the two other cases both CNI and fluconazole dose were rapidly reduced to prevent this complication. Dose-reduction of tacrolimus and/or fluconazole, as well as close monitoring of tacrolimus levels are therefore strongly recommended at the time of azole switch to prevent acute kidney injury [14].

## 4 Conclusion

*Cryptococcocus* sp meningitis is a rare but severe infection in KT patients. Early detection using serum CrAg screening in the most at-risk patients with even minor neurological symptoms should be further investigated. During treatment, close monitoring of renal function is essential due to the nephrotoxicity of L-AmB, but also potential drug-drug interactions between fluconazole and calcineurin inhibitors.

## Declaration of competing interest

There are none.
